# Factors Influencing Formal and Informal Help-Seeking Behavior Among Battered Chinese Women in Beijing, Shanghai, Guangzhou, and Shenzhen

**DOI:** 10.3390/bs15070961

**Published:** 2025-07-16

**Authors:** Ting Zhang, Guan Ren, Hongxi Ge, Huan Zhang

**Affiliations:** School of Government, Beijing Normal University, 19 Xinjiekou Wai St., Beijing 100875, China; 202331240009@mail.bnu.edu.cn (T.Z.); zhugejuncheng@163.com (H.G.)

**Keywords:** battered Chinese women, severity of violence, social support, gender equality awareness, formal and informal, help-seeking behaviors

## Abstract

This study investigates how demographic characteristics, severity of intimate partner violence (IPV), social support, gender equality awareness, and demographic factors (e.g., living with children at home) influence help-seeking behaviors among 2527 IPV-experienced women in Beijing, Shanghai, Guangzhou, and Shenzhen. Drawing on the help-seeking model and conservation of resource theory, the study distinguishes between formal (e.g., police, legal aid) and informal (e.g., family, friends) help-seeking. Logistic regression results reveal that greater violence severity, stronger perceived social support, and higher gender equality awareness significantly increase both formal and informal help-seeking. Notably, living with children is associated with a higher likelihood of seeking formal help, possibly due to increased concerns for children’s safety and the desire to change the abusive environment. While help-seeking behavior is on the rise overall, particularly among women with strong support networks and progressive gender attitudes, structural and cultural barriers remain. The findings underscore the need to improve public education, reduce stigma, and enhance accessibility of support systems. This study contributes to the growing literature on IPV in China and provides evidence for refining policy interventions and service delivery.

## 1. Introduction

In 2021, the World Health Organization (2021) reported that among women ages 15 years and older globally, around 20% to 28% of them had suffered at least one type of intimate partner violence, revealing its widespread prevalence (Sardinha et al., 2024). IPV definitions vary across cultures (Zark & Satyen, 2022); micro-level definitions include physical, sexual, and psychological abuse (Rowlands, 2022), while macro-level definitions add economic control (Strenio, 2021). In China, IPV typically encompasses physical, sexual, and psychological harm (M. Chen & Chan, 2021), consistent with the Anti-Domestic Violence Act (X. Gu et al., 2022). This study adopts the micro-level definition for cultural alignment. Specifically, in China, intimate partner violence (IPV) is officially defined and regulated under the Anti-Domestic Violence Law enacted in 2016. According to the law, domestic violence includes physical, psychological, and other forms of harm, such as beating, verbal abuse, and intimidation, inflicted among family members (J. Chen, 2024). However, the law primarily focuses on physical and sexual and, to a lesser extent, psychological violence, without explicitly addressing other forms such as economic abuse or digital surveillance. This legal framing shapes both public understanding and victims’ help-seeking behaviors and thus provides the structural context for our study.

Despite facing abuse, many women seek help through formal and informal means (Ravi et al., 2022; Wright et al., 2022), influenced significantly by sociodemographic factors (Peraica et al., 2021; Stiller et al., 2025). In China, Confucian-rooted patriarchal norms often normalize IPV and discourage disclosure (G. Y. Gu & Zhong, 2024), reflected in low help-seeking rates (Y. Li et al., 2024). Confucian-rooted gender norms continue to shape attitudes and behaviors in contemporary Chinese society, particularly in the context of family and gender roles. Central to Confucian ideology is the principle of “san cong si de” (三从四德)—the “Three Obediences and Four Virtues”—which dictate that a woman should obey her father before marriage, her husband after marriage, and her son if widowed, while embodying morality, proper speech, modesty, and domestic skills (Wesołowski, 2022). Although China has undergone rapid modernization, Confucian values remain deeply embedded in both institutional frameworks and everyday interpersonal expectations (Chan et al., 2024). These norms emphasize harmony, hierarchy, and the preservation of family unity over individual well-being, often discouraging women from disclosing intimate partner violence or seeking formal help (Y. Li et al., 2024). As a result, Confucianism—while not the only cultural influence—is still a dominant philosophical tradition underpinning family structure and gender relations, particularly among older generations and in less urbanized regions. Understanding the impact of these cultural values is crucial for interpreting help-seeking behaviors in Chinese IPV survivors.

Triggered by the 2000s women’s movement, IPV in China was reframed as a social crime (Begum, 2025). The 2016 Anti-Domestic Violence Act mandated legal responses, including restraining orders and police intervention (J. Lin, 2024), and expanded support services through agencies like the Women’s Federation (Xue et al., 2024). Shelters now offer housing, legal aid, counseling, and medical care (He, 2025), though utilization remains low. Although China’s Anti-Domestic Violence Law (National People’s Congress of China, 2015) mandates the establishment of temporary shelters, the actual number of functioning shelters remains critically low. As of 2023, there were only 1500 domestic violence shelters across the entire country (Women’s Studies Institute of China, 2023), which is far from sufficient given China’s vast population and geographic spread. The limited coverage and under-resourcing of these facilities pose significant barriers to formal help-seeking, particularly in rural or less developed areas. This scarcity underscores the urgent need for expanded infrastructure and sustainable funding to support IPV victims effectively. More importantly, this study investigates Chinese women’s responses to IPV to inform more effective, culturally sensitive support systems. As the four important economic centers in China, Beijing, Shanghai, Guangzhou, and Shenzhen have attracted women from all over the country to work and live here (L. Li & Song, 2024). Therefore, sampling from these four cities has a certain representativeness.

In sum, research on help-seeking behaviors among Chinese women experiencing intimate partner violence (IPV) remains sparse and fragmented. Existing Chinese studies are often regionally limited, based on small samples, and tend to treat IPV as a culturally sensitive issue, leading to cautious and indirect discussions (Zheng et al., 2024; Yuan et al., 2024). Most of these studies focus solely on whether women seek help, without distinguishing between formal (e.g., police, legal services) and informal (e.g., family, friends) help-seeking behaviors. Moreover, limited empirical attention has been paid to how key factors such as violence severity, social support, and gender equality awareness shape help-seeking decisions. Instead, past Chinese research has primarily emphasized individual or family-level characteristics like education or marital status (Y. Li et al., 2024). This lack of comprehensive data and theoretical development underscores the need for more robust, large-scale, and theory-informed studies.

In response, the present study aims to fill this gap by examining a large sample of 2527 IPV-experienced women across four major urban centers in China. We go beyond basic demographic predictors and incorporate multiple influencing factors—including severity of IPV, perceived social support, and gender equality awareness—thereby offering a more nuanced understanding of Chinese women’s help-seeking behaviors in both formal and informal domains.

## 2. Literature Review and Hypotheses Development

Research on help-seeking among battered women has identified a complex interplay of factors influencing whether and how women seek assistance when facing intimate partner violence (IPV). These include demographic characteristics, types and severity of violence, levels of social support, and awareness of gender equality. This study draws on the help-seeking model proposed by Liang et al. (2005), which conceptualizes help-seeking as a dynamic, multi-stage process involving the recognition of abuse, the decision to seek help, and the selection of help sources. The framework provides a comprehensive lens through which to understand how both internal and external factors interact to shape help-seeking behavior.

### 2.1. Demographic Characteristics

Demographic variables are among the most commonly examined predictors of help-seeking behaviors. However, the findings are mixed and often context-dependent. Regarding age, some studies suggest that older women are more likely to seek help due to longer exposure or greater awareness of abuse consequences (Nobels et al., 2024). In contrast, other research finds that younger women are more proactive in seeking support, particularly due to digital literacy, changing gender norms, or increased access to information (E. Y. Y. Kim et al., 2024). Given these varying results, this study proposes the following:

**Hypothesis** **1.** *The battered women’s age is negatively related to their help-seeking (informal and formal). Younger battered women are more likely to seek help.*

Education is another important factor. Higher educational attainment has been linked to increased help-seeking, especially formal help, as educated women may better recognize abuse, understand legal rights, and possess the confidence to engage with formal institutions (Morishita et al., 2024).

**Hypothesis** **2.** *The battered women’s education background is positively related to their help-seeking (informal and formal). Battered women who have higher education backgrounds are more likely to seek help.*

Similarly, income is positively associated with help-seeking behavior. Women with greater economic resources are more likely to access professional services and legal assistance due to reduced financial dependence on the abuser and increased autonomy (Morishita et al., 2024).

**Hypothesis** **3.** *The battered women’s income is positively related to their help-seeking (informal and formal). Battered women who have higher incomes are more likely to seek help.*

Employment status also plays a role (Marwitz et al., 2024). Women who are employed may feel more empowered and independent, making it easier to take steps toward seeking help, especially from formal institutions.

**Hypothesis** **4.** *The battered women’s employment status is positively related to their help-seeking (informal and formal). Battered women who have a job are more likely to seek help.*

In addition, living with children can influence women’s help-seeking behaviors. Some women report seeking help primarily to protect their children, rather than to end the abusive relationship (Badenes-Sastre et al., 2024). Research also suggests that the presence of children increases perceived responsibility and urgency for action (Yuan et al., 2024).

**Hypothesis** **5.** *The battered women’s living with children status is positively related to their help-seeking (informal and formal). Battered women who are living with their children are more likely to seek help.*

### 2.2. Violence Severity and Type

The severity and type of IPV significantly shape how and whether women seek help. While some studies argue that severe physical abuse prompts women to pursue formal interventions (Zhang et al., 2024), others find that such abuse may lead primarily to informal support-seeking due to fear, stigma, or institutional distrust (Ravi et al., 2024). Sexual coercion tends to push women toward legal channels, given its association with serious harm and social condemnation (Kashino, 2025). In contrast, psychological abuse is less visibly damaging and often results in women turning to informal networks for emotional support (Gupta et al., 2024).

Despite these variations, studies consistently find that more severe abuse, regardless of type, increases the likelihood of help-seeking, even if the pathway (formal vs. informal) varies by violence type (Leonardsson & San Sebastian, 2017; Y. Li et al., 2024). Based on this literature:

**Hypothesis** **6.** *The severity of physical, psychological, and sexual intimate partner violence is positively related to battered women’s help-seeking (informal and formal).*

### 2.3. Social Support and Gender Equality Awareness

Social support is widely recognized as a facilitator of help-seeking. Women embedded in strong social networks often feel safer and more empowered to disclose abuse and access help (Cho et al., 2021; D’Urso et al., 2024). Social ties provide emotional encouragement, material assistance, and information about available services, all of which reduce the perceived barriers to help-seeking. This aligns with conservation of resources (COR) theory (Hobfoll, 1989), which suggests that individuals strive to retain, protect, and restore valuable resources. From this perspective, help-seeking becomes an effort to recover lost or threatened resources, such as personal safety, psychological well-being, or custody of children.

**Hypothesis** **7.** *The battered women’s social support is positively related to their help-seeking (informal and formal).*

Gender equality awareness is another increasingly recognized factor influencing IPV responses. Women who are aware of gender-based rights and who reject traditional patriarchal norms are more likely to interpret abusive behavior as unacceptable and take steps to seek help (Green et al., 2024). In China, gender equality education has been promoted through national curricula and legal reforms (Guo, 2019), potentially enhancing women’s ability to identify abuse and pursue intervention.

**Hypothesis** **8.** *The battered women’s gender equality awareness is positively related to their help-seeking (informal and formal).*

By integrating Liang et al.’s (2005) multi-stage model of help-seeking and Hobfoll’s (1989) COR theory, this study aims to construct a comprehensive framework for understanding battered women’s help-seeking behaviors in the Chinese context. Liang’s model highlights the psychological decision-making stages, while COR theory emphasizes the role of resource protection and restoration. The interaction of personal demographics, the nature of the violence experienced, social environments, and cognitive awareness collectively shape whether, when, and how women seek help.

## 3. Method

### 3.1. Sample

This study employed a multistage stratified random sampling strategy in four major Chinese cities—Beijing, Shanghai, Guangzhou, and Shenzhen—from January to December 2024. In each city, 50 residential communities were randomly selected from the list provided by the local government. Within each selected community, 100 adult women were randomly recruited with the assistance of local community committee staff, yielding a total sample of 5000 women per city and 20,000 participants overall. To ensure privacy, safety, and comfort, data collection did not take place in respondents’ homes. Instead, participants were invited to local police substations, where trained female data collectors conducted the survey in private rooms with the support of community police officers. Prior to participation, all women were individually approached and informed about the study. Informed verbal and electronic consent were obtained from each participant. Fortunately, all women agreed to take part and completed the full process. As appreciation for their time and contribution, each participant received a 20 RMB incentive upon completing the survey.

Among the 20,000 respondents, 3425 women (17.1%) reported experiencing at least one form of intimate partner violence (IPV) in their lifetime. Of these, 2527 women who reported seeking at least one type of formal or informal help were included in the main analysis. Women who experienced IPV but did not seek help (*n* = 898) were excluded. All data collection was conducted using a 30 min anonymous electronic questionnaire administered via tablet devices. Our data collectors were trained female graduate students in social work, supervised by professionals specializing in domestic violence intervention. The study was approved by the Ethics Committee of Beijing Normal University (SSDPP-HSC 2022010) on 2 November 2022 and officially supported by the municipal governments of the four cities involved.

### 3.2. Measures

#### 3.2.1. Help-Seeking Behavior (Dependent Variable)

Help-seeking behavior, the dependent variable, was categorized into four formal and one informal type (J. Y. Kim & Lee, 2011). Formal help-seeking was measured using four binary questions (0 = no, 1 = yes) about whether participants had, in the past year, (1) reported IPV to the police, (2) contacted legal services or courts, (3) sought medical assistance, or (4) contacted domestic violence shelters. Informal help-seeking was assessed with one binary item: whether participants had sought help from friends, family, or neighbors. Each item was analyzed separately.

#### 3.2.2. Demographic Characteristics (Independent Variable)

Demographic variables were coded based on contextually relevant criteria and prior empirical studies. Age was dichotomized as 0 (under 30) or 1 (30 and older), based on research identifying age 30 as a key transition point in women’s lives, particularly in terms of marital, reproductive, and occupational expectations (Brown et al., 2017). Education was coded as 0 (less than high school) and 1 (high school or above). This classification reflects China’s national education policy, where high school marks the upper limit of compulsory education, and attainment beyond this level is typically associated with greater socioeconomic status and awareness of rights (Xiong, 2019). Monthly income was coded as 0 (below 5000 yuan) or 1 (5000 yuan and above), based on the 2018 adjustment of China’s personal income tax threshold, which raised the minimum taxable income from 3500 to 5000 yuan. This threshold has since become a benchmark for defining economic vulnerability and middle-income status in national policy discussions (Peng & Xiao, 2019). Employment status was coded as 0 (unemployed) and 1 (employed), following standard practices in prior studies of IPV and economic dependence (e.g., X. Wang & Zhang, 2016). Employment is treated as a key protective factor affecting help-seeking and autonomy. Living with children was coded as 1 (co-residing with one or more children) and 0 (not co-residing). This binary coding reflects caregiving responsibilities and household composition, which may influence both women’s exposure to IPV and their motivation to seek help.

#### 3.2.3. Social Support (Independent Variable)

Social support was measured using the Perceived Social Support Questionnaire (Kliem et al., 2015), which shows high internal consistency (Cronbach’s alpha = 0.90) in the Chinese version (M. Lin et al., 2019). The scale includes six items: (1) I feel understood, cared for, and secure by others; (2) I have a reliable person to turn to for help; (3) I can easily borrow things from neighbors or friends; (4) I have several people I enjoy spending time with; (5) I can ask friends or family to handle important matters when I am sick; (6) I know who to turn to for support when I am sad. Responses were rated on a 5-point scale (1 = totally disagree, 5 = totally agree), with scores ranging from 5 to 25.

#### 3.2.4. Gender Equality Awareness (Independent Variable)

Gender equality awareness was measured using a 5-item scale developed by Dong (2018), with strong reliability (Cronbach’s alpha = 0.84). Each item was rated on a 5-point scale (1 = strongly disagree, 5 = strongly agree), with scores ranging from 5 to 25. Participants rated statements including the following: (a) Men should prioritize careers, while women should focus on family. (b) Men are inherently more capable than women. (c) A good marriage is more important for women than a career. (d) Female employees should be laid off first during economic downturns. (e) Husbands and wives should share housework equally. While unlike the other four items, the fifth item, “Husbands and wives should share housework equally,” was positively worded. To ensure consistency in scoring, the first four items were reverse-coded so that higher total scores indicated stronger gender equality awareness.

#### 3.2.5. Severity of Physical, Sexual, and Psychological Violence (Independent Variable)

IPV severity was measured using the Revised Conflict Tactics Scale (CTS2; Straus et al., 1996), which includes three subscales: physical assault (12 items), psychological aggression (8 items), and sexual coercion (7 items). Each subscale assesses the frequency of a specific type of violence experienced from an intimate partner during the past 12 months. Representative sample items are as follows: for physical assault, “My partner pushed or shoved me”, “My partner punched or hit me with something that could hurt”; for psychological aggression, “My partner insulted or swore at me”, “My partner destroyed something belonging to me”; and for sexual coercion, “My partner used force (like hitting or holding down) to make me have sex”, “My partner insisted on sex when I did not want to, without using physical force.” Participants responded on a 5-point frequency scale ranging from 0 (never) to 4 (every day). Frequency scores were summed within each subscale: physical assault (0–48), psychological aggression (0–32), and sexual coercion (0–28). The mean scores for each subscale reflect the severity of violence experienced, with higher scores indicating greater severity. The original CTS2 showed high internal consistency (α = 0.86 for physical assault, 0.87 for sexual coercion, and 0.79 for psychological aggression; Straus et al., 1996). In the current study, the subscales demonstrated similarly acceptable internal reliability (Cronbach’s α > 0.75 for all three subscales).

### 3.3. Data Analysis

As a typical cross-sectional survey design, data were analyzed using SPSS 24.0. Descriptive statistics were computed for all variables, and help-seeking behaviors were assessed as five separate variables: police reports, medical agencies, legal services, shelters, and family/neighbors. Each was analyzed independently using chi-square and Spearman tests for bivariate correlations. No collinearity was found, as the conditional index score, VIF, tolerance, and eigenvalue were within acceptable ranges. Logistic regression was used to examine correlations between variables, with demographic data entered in Model 1, followed by violence severity in Model 2, social support in Model 3, and gender equality awareness in Model 4.

## 4. Results

### 4.1. Descriptive Statistics

As shown in Table 1, 54.3% of participants were under 30 years old, with over 70% having completed high school or more and nearly 65% earning at least 5000 yuan monthly. Most (78.5%) were employed, while 55.1% did not live with children. Violence severity ranged from none to daily (did not report in the table), with mean scores for physical violence (M = 21.32, SD = 3.95), sexual coercion (M = 10.3, SD = 3.1), and psychological aggression (M = 19.2, SD = 3.7). Social support (M = 15.1, SD = 2.51) and gender equality awareness (M = 17.3, SD = 4.09) were also assessed. Regarding help-seeking, 97.3% sought help from family or neighbors, 50.6% contacted the police, 24.9% used legal services, and fewer used shelters (4.7%) or medical services (15.4%).

### 4.2. Association Among Independent and Dependent Variables

Chi-square tests (Table 2) revealed that younger participants (under 30) were more likely to contact the police (71.3% vs. 28.7%, χ^2^ = 5.013, *p* = 0.012), as were those with higher education (61.2% vs. 38.8%, χ^2^ = 2.109, *p* = 0.003) and those living with children (85.7% vs. 14.3%, χ^2^ = 4.213, *p* = 0.022). Similar trends were observed for contacting legal services. However, no demographic differences were found among women who approached medical agencies, shelters, or family/friends. Spearman test results (Table 3) showed that police help-seeking was linked to physical violence, sexual coercion, and gender equality awareness, while medical help-seeking was associated with physical violence and sexual coercion. Legal help-seeking was related to physical violence, sexual coercion, and gender equality awareness, while informal help-seeking was linked to physical violence, sexual coercion, and social support. No internal correlation was found among independent variables.

### 4.3. Factors Contributing to Battered Chinese Women’s Help-Seeking Behaviors

As explained in the data analysis section, our analysis required data entry in four steps. Table 4 and Table 5 show the result of these analyses.

#### 4.3.1. Contacting Police

In Model 1, demographic variables such as age (negatively), education (positively), and living with children (positively) predicted police contact. In Model 2, the severity of physical and sexual violence was positively associated with seeking police assistance. Model 3 showed no correlation between social support and police contact but did increase the model’s explained variance. Finally, in Model 4, gender equality awareness was significantly and positively related to contacting the police, even after controlling for demographics, violence severity, and social support.

#### 4.3.2. Contacting Medical Agencies

No demographic variables were correlated with contacting a medical agency (Model 1), but the severity of physical and sexual violence positively predicted whether participants sought medical assistance (Model 2). Model 3 increased the model’s explained variance but didn’t show any correlation between social support and seeking medical assistance. Model 4 played a similar role as Model 3.

#### 4.3.3. Contacting a Legal Services Agency

Model 1 shows that age (negatively), educational background (positively), and monthly income (positively) were related to the behavior of contacting legal services. According to the results in the other three models, level of physical violence, sexual coercion, and gender equality awareness were positively correlated with contacting legal assistance.

#### 4.3.4. Contacting Shelters

No factors were significantly related to the behavior of contacting a shelter.

#### 4.3.5. Contacting Friends, Family, or Neighbors

In Model 2, the severity of physical and sexual violence was positively associated with seeking informal assistance. Model 3 and Model 4 significantly increased the model’s strength and showed that social support was positively associated with the likelihood that battered women would approach an informal resource.

In sum, based on the results, several hypotheses proposed in this study were confirmed. Hypotheses 1–5, which posited that younger age, higher education, higher income, employment, and co-residence with children would be positively associated with help-seeking, were largely supported—particularly for formal help-seeking behaviors such as contacting the police and legal services. Specifically, age, education, income, and child co-residence significantly predicted help-seeking from these formal sources. Hypothesis 6 was partially confirmed: physical and sexual violence severity were strong predictors of both formal and informal help-seeking, while psychological violence did not significantly influence any help-seeking behavior. Hypothesis 7 was supported for informal help-seeking only; higher levels of social support were associated with contacting friends, family, or neighbors, but not formal agencies. Finally, Hypothesis 8 was partially supported: gender equality awareness emerged as a consistent and significant predictor of formal help-seeking across multiple models, highlighting the role of internal attitudes in shaping action. Overall, the findings validate the theoretical model that integrates individual, relational, and sociocultural factors in understanding battered women’s help-seeking behaviors in China.

## 5. Discussion

Regarding formal and informal assistance resources, the majority of participants (97.3%) sought help from their families or neighbors. Additionally, 50.6% contacted the police in the past year, and 24.9% reached out to legal services or lawyers for assistance with IPV issues. The least used resources were domestic shelters (4.7%) and medical agencies (15.4%). Consistent with previous research (Sharifi et al., 2024), Compared to battered Chinese women in the past, who displayed higher endurance of IPV (Huang et al., 2024), women in this study sought help at higher rates. Despite this progress, traditional Chinese cultural norms, influenced by Confucianism, still hinder many women from reporting IPV outside the family (Cho et al., 2021). Nevertheless, a significant number of participants in this study sought legal services, indicating a shift away from these barriers. This aligns with other research showing increased utilization of legal services after the implementation of the Anti-Domestic Violence Act (e.g., Kui, 2024), which clarifies law enforcement responsibilities and encourages women to approach police and legal services. The low use of shelters may be due to misunderstandings of their role, as it was found that battered women viewed shelters as shameful and unsafe (Miao & Westmarland, 2025). To address this, it is essential to improve awareness of shelter services and reduce the stigma surrounding their use.

In this study, age, educational background, and co-residence with children were significant predictors of formal help-seeking behaviors. Consistent with prior research (e.g., Smith, 2024), younger women were more likely to seek help, aligning with findings by Y. Wang (2019) that younger Chinese women show less endurance of IPV. Women with higher education were also more likely to access police or legal services, as supported by previous studies (Duterte et al., 2008; Fanslow & Robinson, 2010; Stephens & Eaton, 2020). This reflects a broader trend in China where more educated women are less constrained by patriarchal norms and more inclined to seek formal assistance (X. Wang et al., 2019). Additionally, monthly income was positively associated with contacting legal services, likely due to the high cost of legal services in China (Zhao & He, 2024). To improve access for low-income women, expanding public attorney services and establishing nonprofit legal aid could be key strategies.

Consistent with numerous studies, the severity of physical and sexual violence was a significant predictor of both formal and informal help-seeking (e.g., Domenech del Rio & Sirvent Garcia del Valle, 2019). Such violence often leads to severe physical harm, triggering a survival instinct that motivates victims to seek protection and escape (Leonardsson & San Sebastian, 2017). In contrast, the lack of clear understanding of psychological abuse may contribute to its limited impact on help-seeking, as Y. Li et al. (2024). noted that battered women often fail to recognize its severity. This could explain the weak correlation between psychological violence and help-seeking in the current study.

Additionally, gender equality awareness and social support emerged as predictors of seeking formal and informal help, consistent with previous research (D’Urso et al., 2024). Gender equality education, which has been integrated into China’s elementary education for decades (Guo, 2019), has been linked to increased awareness and help-seeking behaviors. This suggests that both gender equality awareness and help-seeking behaviors among battered Chinese women will continue to rise.

The key finding of this study is the significant correlation between the presence of children in the household and help-seeking behaviors among battered women, indicating a shift in China away from the traditional family norm where women endure domestic violence for the sake of their children. This aligns with previous studies, such as Yuan et al. (2024), who found that women with children are more likely to seek help to protect their children, and Zheng et al. (2024), who concluded that battered women are more inclined to seek support when their children are affected by IPV. Research has consistently shown that children exposed to IPV face increased risks of physical harm and mental health issues like PTSD (Maji, 2018).

In response to these risks, the Chinese government has implemented comprehensive child protection legislation in the past decade, such as the “Anti-domestic violence law” and related regulations aimed at preventing family harm and safeguarding children’s welfare (J. Chen, 2024). These policies enhance legal protections for children exposed to domestic violence and promote coordinated social services for affected families. Such governmental measures are critical in empowering battered women to seek formal help, as they alleviate fears related to child custody and welfare that historically discouraged reporting.

Moreover, more comprehensive protection laws have contributed to women’s greater awareness of the detrimental effects of IPV on children’s development, reinforcing the decision to leave violent households in pursuit of safer environments for their children. This trend reflects a positive social transformation that aligns with global movements toward child-centered approaches in IPV intervention (X. Gu et al., 2022). Therefore, the protective acts for children not only serve to directly shield minors but also indirectly facilitate help-seeking behaviors among mothers, indicating an important policy impact in the fight against domestic violence in China.

### 5.1. Practical Implications

The findings of this study underscore the positive impact of existing policies, such as the Anti-Domestic Violence Law and comprehensive child protection legislation, in encouraging help-seeking behaviors among battered women in China. These laws have clarified legal responsibilities, enhanced protections, and promoted service coordination, contributing to increased utilization of formal support systems (J. Chen, 2024; G. Y. Gu & Zhong, 2024). However, challenges remain. The low utilization of shelters suggests a need to expand shelter capacity and improve public awareness to reduce stigma and misconceptions about these resources (Miao & Westmarland, 2025). Furthermore, legal services remain costly, limiting access for low-income women, highlighting the necessity to broaden nonprofit legal aid and publicly funded attorney services (Zhao & He, 2024).

Expanding community-based interventions and gender equality education can further empower women, fostering social support networks essential for effective help-seeking (D’Urso et al., 2024). Inter-agency collaboration between police, legal systems, social services, and healthcare providers should be strengthened to offer comprehensive, victim-centered responses. Finally, tailored public education campaigns are needed to challenge traditional cultural norms that still hinder reporting and to raise awareness about the harmful effects of psychological abuse, an often overlooked IPV form (Y. Li et al., 2024).

In sum, while current policies provide a foundation for protecting IPV victims, enhancing shelter availability, legal aid accessibility, and multi-sector cooperation will be crucial for addressing remaining gaps and ensuring more effective, inclusive support for battered women in China.

### 5.2. Limitation

While this study provides valuable insights into the help-seeking behaviors of battered women in Beijing, Shanghai, Guangzhou, and Shenzhen, several limitations should be noted. Compared to the total number of women in China, the sample size was relatively small, and due to funding constraints, data was only collected in these four big cities. However, given these cities statuses as major economic hubs, many women from diverse cultural backgrounds across China have migrated there. Despite this, the higher cost of living in these cities may result in a sample with higher educational levels and income compared to women in other regions, which limits the generalizability of the findings to less economically developed areas. Additionally, the social support questions focused only on friends and neighbors, excluding questions related to national policies or organizations. Finally, the study did not examine the influence of child abuse on battered women’s help-seeking behaviors, which warrants further exploration in future research.

## 6. Conclusions

Very few studies have explored help-seeking behaviors among battered Chinese women, and this research seeks to address that gap. The findings reveal that improvements in education, income, and gender equality awareness have made younger women more likely to seek help from formal resources. Additionally, the severity of physical and sexual violence significantly influences their decision to seek assistance, particularly from formal channels. However, shelters were the least utilized resource, highlighting a need for further investigation into this phenomenon. In addition, public education programs are crucial for helping individuals recognize the signs of all types of abuse, especially psychological abuse. Efforts should focus on increasing awareness of IPV and available support resources, enabling women to access the help they need when faced with violence.

## Figures and Tables

**Table 1:** Demographic characteristic of participants (*N* = 2527).

	Total		Police		Medical Agency		Legal Services		Shelter		Families or Neighbors	
	(*N* = 2527)		(*n* = 1278)		(*n* = 388)		(*n* = 628)		(*n* = 120)		(*n* = 2458)	
	*n* (%)		*n* (%)		*n* (%)		*n* (%)		*n* (%)		*n* (%)	
Age												
Younger than 30	1372	54.3	912	71.3	119	30.8	424	67.5	86	72.0	1273	51.8
30 and older than 30	1155	45.7	366	28.7	269	69.2	204	32.5	34	28.0	1185	48.2
Education												
Below high school	746	29.5	496	38.8	92	23.7	134	21.4	26	22.0	1074	43.7
High school or beyond	1781	70.5	782	61.2	296	76.3	494	78.6	94	78.0	1138	46.3
Monthly income												
Below 5000 yuan	889	35.2	344	26.9	75	19.2	204	32.4	31	26.0	917	37.3
5000 yuan or more	1638	64.8	934	73.1	314	80.8	425	67.6	89	74.0	1541	62.7
Employment												
Unemployed	543	21.5	303	23.7	149	38.5	168	26.8	28	23.0	1015	41.2
Employed	1984	78.5	975	76.3	239	61.5	460	73.2	92	77.0	1443	58.8
Living with children at home												
No	1392	55.1	183	14.3	228	58.9	170	27.1	74	62.0	1280	52.5
Yes	1135	44.9	1095	85.7	160	41.1	458	72.9	46	38.0	1178	47.5

**Table 2:** Chi-square test results.

	1	2	3	4	5	6	7	8	9
	χ^2^	χ^2^	χ^2^	χ^2^	χ^2^	χ^2^	χ^2^	χ^2^	χ^2^
2	1.125								
3	1.543	2.136							
4	2.236	2.247	2.212						
5	2.118	2.358	2.134	3.221					
6	5.013 *	2.109 *	2.245	3.932	4.231 *				
7	2.345	5.124	2.876	2.987	4.012	3.234			
8	3.921 *	2.567 *	2.256 *	4.243	3.222 *	4.256	3.712		
9	2.834	4.832	1.234	1.765	2.243	1.654	2.235	2.246	
10	4.912 *	1.945	2.013	2.256	1.234	2.245	1.923	1.245	2.234

Note. 1 = age; 2 = education; 3 = income; 4 = employment status; 5 = living with children at home; 6 = seeking help from police; 7 = seeking help from medical agency; 8 = seeking help from legal services; 9 = seeking help from shelter; 10 = seeking help from family, neighbors, or friends. * *p* < 0.05.

**Table 3:** Spearman test results.

	1	2	3	4	5	6	7	8	9	10	11	12	13	14
11	0.532	0.673	0.283	0.132	0.932	0.673 *	0.842 *	0.302 *	0.213	0.204 *				
12	0.204	0.774	0.140	0.131	0.383	0.240	0.832	0.218	0.246	0.135	0.439			
13	0.663	0.688	0.240	0.185	0.267	0.325 *	0.672 *	0.202 *	0.302	0.224 *	0.345	0.126		
14	0.562	0.272	0.409	0.320	0.312	0.213	0.242	0.153	0.404	0.409 *	0.412	0.272	0.122	
15	0.694	0.197	0.482	0.412	0.862	0.486 *	0.562	0.783 *	0.442	0.742	0.262	0.139	0.213	0.139

Note. 1 = age; 2 = education; 3 = income; 4 = employment status; 5 = living with children at home; 6 = seeking help from police; 7 = seeking help from medical agency; 8 = seeking help from legal services; 9 = seeking help from shelter; 10 = seeking help from family, neighbors, or friends; 11 = physical violence; 12 = psychological assault; 13 = sexual coercion; 14 = social support; 15 = gender equality awareness. * *p* < 0.05.

**Table 4:** Logistic regression analysis of variables predicting formal help seeking (*n* = 2527).

	Police				Medical Agency				Legal Services				Shelter			
Variables	1	2	3	4	1	2	3	4	1	2	3	4	1	2	3	4
*Demographics*																
Age	−0.925 *	−1.012 *	−0.972 *	−1.182 *	−1.023	−1.132	−1.285	−0.986	−0.873 *	−0.823 *	−0.938 *	−0.986 *	−0.792	−0.882	−0.993	−0.664
Education	2.125 *	2.042 *	2.246 *	2.572 *	2.772	2.472	3.019	1.993	3.435 *	3.174 *	3.473 *	3.123 *	1.033	1.020	1.164	1.191
Income	1.776	1.763	1.603	2.001	4.993	4.763	3.988	4.083	0.893 *	1.112 *	0.938 *	0.947 *	0.164	0.303	0.237	0.193
Employment	2.209	2.115	2.111	1.998	2.982	2.205	2.015	2.683	0.796	0.883	0.895	0.826	0.183	0.264	0.239	0.213
Living with children at home	1.093 *	1.054 *	1.022 *	1.012 *	1.663	1.383	1.866	1.773	2.112 *	2.552 *	2.176 *	1.992 *	1.033	1.109	1.093	1.974
*Severity of violence*																
Physical		3.226 *	3.408 *	3.010 *		2.197 *	1.993 *	2.015 *		3.112 *	3.129 *	2.992 *		3.021	2.209	3.015
Psychological		2.973	2.518	2.004		1.930	1.663	1.794		2.187	2.130	2.362		2.204	2.216	2.209
Sexual		3.254 *	4.121 *	4.001 *		1.773 *	1.892 *	1.695 *		1.684 *	1.727 *	1.609 *		2.894	2.552	3.103
*Others*																
Social support			2.098	2.096			0.914	0.903			2.128	2.231			1.174	1.232
Gender equality awareness				3.045 *				1.670				1.865 *				1.063
Constant	0.209	0.365	0.472	0.762	2.094	3.012	1.874	2.995	2.208	2.240	1.994	2.054	0.017	0.203	0.210	0.214
Model χ^2^	8.204	10.004	12.274 *	13.174 *	7.573	7.854	9.775 *	9.987 *	5.285	5.398	5.993 *	7.884 *	7.745	8.143	8.276	8.998
*Df*	6	9	10	11	6	9	10	11	6	9	10	11	6	9	10	11
△χ^2^		0.967	6.864 *	8.702 *		0.884	2.674 *	3.093 *		1.988	1.476 *	1.103 *		0.373	0.205	0.894

* *p* < 0.05.

**Table 5:** Logistic regression analysis of variables predicting informal help seeking (*n* = 2527).

	Friends, Family, or Neighbors			
Variables	1	2	3	4
*Demographics*				
Age	−1.566 *	−1.634	−1.473	−1.164
Education	1.993	1.774	1.693	1.447
Income	2.054	2.167	2.198	2.204
Employment	2.020	2.113	1.993	2.265
Living with children at home	1.103	1.284	1.113	1.456
*Severity of violence*				
Physical		1.275 *	1.178 *	1.296 *
Psychological		1.020	1.304	1.296
Sexual		0.997 *	0.873 *	0.664 *
*Others*				
Social support			2.472 *	2.572 *
Gender equality awareness				1.953
Constant	1.103	2.009	2.104	2.209
Model χ^2^	6.992	7.295	8.375 *	9.993 *
*Df*	6	9	10	11
△χ^2^		1.434	1.875 *	2.005 *

* *p* < 0.05.

## Data Availability

The datasets presented in this article are not readily available because of the ethical limitation. Due to the nature of this research, participants of this study did not agree for their data to be shared publicly, so supporting data is not available.
